# Recent Cases of Tetanus in the British Expeditionary Force

**Published:** 1918-01

**Authors:** 


***Recent Cases of Tetanus in the British Expeditionary Force.*** By Sir William B. LEISHMAN, Colonel, R. A. M. S., and Major A. B. SMALLMAN, R. A. M. C. Abs. from the *Lancet*, Jan. 27, 1917.

L. and S. present a report on the value of serum treatment for tetanus as observed in France. They collected their data and tabulated them in such a way as to correlate their work with that of Sir David Bruce for tetanus cases in home hospitals (see p. 184).

The statistics presented are based upon cases occurring in the British forces in France between July 1 and Oct. 31, 1916.

Of the 160 cases reported, 118 died and 42 recovered, a case-mortality of 73.7 o/o. A group of 179 cases reported the previous year by L. indicated a mortality of 78.2 o/o. There seems, then, to have been very little improvement during the year in the treatment of tetanus. The mortality in cases treated in England for the two years, as reported by Bruce, was considerably lower, 57.7 o/o for 1915 and 49.2 o/o for 1916. Only a slight improvement is indicated on both sides of the channel, but it appears that the mortality is 25 o/o higher in France than in England. This fact is, of course, easily explained by the fact that severe wounds which are most liable to tetanus and complications, never reach England.

The authors can give no precise information as to conditions under which there is a high incidence of tetanus, but they are convinced that it is more prevalent when military operations occur in wet and muddy weather. Similarly they can present no statistics as to preventive injections. They state that this practice is almost universal in all the armies, including the enemy's and that as a result the medical services have to deal with hundreds of cases instead of thousands.

Of the 113 fatal cases, 62 (55 o/o) were from multiple wounds, 98 (87 o/o) were from severe wounds, 88 (78 o/o) were complicated with severe sepsis, and 61 (54 o/o) with gas gangrene. The authors call special attention to the prevalence of this last disease, which they believe is largely responsible for the high percentage of mortality in cases of tetanus at the front.

It was also observed that there was a slightly higher mortality (6 o/o) from body and head cases, than from those of the limbs, due perhaps to the shorter distance along the nerves to the spinal column.

The statistics on the period of incubation in general confirm those presented by Bruce for the home hospitals. The incubation period for the fatal cases was slightly shorter, on the average, than for those that recovered (10.7 days for fatal cases, 14 days for cases that recovered). The highest incidence of the disease occurred upon the 8th day instead of the 11 th as in England. 2 cases with an incubation period of only 2 days were reported. The cases of delayed tetanus were much fewer than in the home hospitals. The longest periods of incubation were 52 and 90 days : both cases were fatal, one resulting from a slight abrasion, the other from an operation. In all there were 13 cases of late tetanus (with an incubation period of 21 or more days). Among these there were 8 deaths (average incubation period of 36.3 days) and 5 recoveries (average incubation period 29.4 days).

As to the effect of operation upon tetanus, it was observed that when the operation was performed either before or after the onset of the tetanus, the number of the cases were too few, and complicating considerations too numerous to make the statistics reliable. The authors conclude on this point that it is wise to give a sufficient prophylactic dose a short time before an operation, but that when tetanus has once set in, any severe disturbance of the wound, or any severe operation should be avoided until the signs of abatement are observed and a sufficient amount of antitoxin has been administered.

On the disputed points concerning the preventive use of antitoxin, the authors note that the percentage of mortality among cases in which the injection was made within 24 hours of the injury was 62.7 o/o; whereas among cases in which the injection was made later, the mortality was 86.9 o/o. They remark, however, that cases in which the injection is delayed are generally those which are exposed in " no man's land " for several days before they are rescued, and that such cases are usually septic. They also note that the incubation period in cases with delayed injections is shorter than in those treated the first day. Also that recovery after delayed injections is more common when the preventive dose is large (at least 915 units).

Of the 15 cases in which no preventive dose had been given, 9 (60 o/o) died. Many of these, however, were very slight injuries.

*Therapeutic Use of Serum.* — The authors took special pains in collecting and tabulating statistics on this point. They think their results valuable by way of suggestion, although they regret that the present confusion in applying treatment, makes any accurate conclusions impossible.

*Dosage.* — Like Bruce they find a much higher percentage of mortality for cases in which the total dosage was small (less than 20,000 units) than in cases treated by a total dose of more than 20,000 units, a balance in favor of the higher dose amounting to 20 o/o. Similarly Bruce's tables show a favorable balance of 25 o/o. The authors think that no fallacies in the statistics can account for this very great advantage derived from the higher dosage.

*Method of Injection.* — As was the case in Bruce's report, the statistics show no certain advantage to be derived from *intrathecal injections*. When this route alone was chosen the mortality was 84 o/o. If the intrathecal injections were supplemented by others, the results were generally better. The lowest percentage of mortality in cases treated by intrathecal injections was found in those in which the intramuscular method was also used; this percentage was about 51 o/o. It appears, therefore, that if the intrathecal route is used at all it should be combined with some other.

None of the 7 cases treated only by *intravenous* injections recovered. When this method was combined with others, the most favorable results were again obtained when the intramuscular route was also employed.

Of all the routes, when used alone, the *subcutaneous* seems to have given the best results, a mortality of only 55 o/o. 27 cases were so treated. In combinations with other routes it was much less successful, except when it was used with the intramuscular route.

The *intramuscular* route, combined with others, gave the most favorable results. Of the total number of cases in which it was used (32) there was a mortality of 59 o/o, the lowest recorded for any route. It was used alone in only 4 cases, and of these three died, a mortality of 75 o/o. If, however, these 4 cases are grouped together with the 8 cases reported by Bruce, it is interesting to note that for the twelve cases in which we know the intramuscular route to have been used alone we have a mortality of only 35.3 o/o, the lowest reported for any of the routes when used alone.

The authors remind us that any conclusions based upon statistics of this nature must be very unreliable. The accumulative effect of them, however, is to throw considerable doubt upon the value of the intrathecal and intravenous routes and, on the other hand, to reestablish faith in the intramuscular and subcutaneous injections.

These conclusions are further supported by grouping the various routes with a view to determining the effect of the size of doses upon the efficacy of any method of treatment. In the case of the intrathecal and intravenous routes the tables show that the size of the doses seems to make little difference in the mortality. In the case of the intramuscular and subcutaneous routes however, the correspondence between the size of the doses and the number of recoveries is very marked. Thus the suspicion against the intravenous and intrathecal methods of injection is strengthened, and the value of the subcutaneous and intramuscular channels emphasized.

*Conclusions.* — The authors agree with the recommendation of the Tetanus Committee in their revised Memorandum (see p. 183) that the intravenous route should be abandoned. They dispute, however, the recommendation made by the Committee in favor of the intrathecal route. They think that theoretically the value of antitoxin in the spinal column for tetanus is far from certain, as the purpose of the injection is very different from that in the case of meningitis. They also contend that this method of treatment is attended by serious dangers, such as the contamination of the needle track, the irritating action of the preservative antiseptics mixed with the serum, and anaphylactic shock.

The authors, however, admit that in individual cases doctors report very gratifying results from the intrathecal method of injection, and they note that it formed part of the treatment in 27 cases which recovered.

If it is used, they recommend the following precautions : perfect antisepsis to prevent contamination of the spinal canal, the omission of preservative antiseptics from the serum, and the removal of not more than 10 to 15 cc. of the spinal fluid. A desiccated antitoxin meets these requirements best, and it can be rapidly prepared under sterile conditions in the desired concentration.

The superiority of the subcutaneous and intramuscular routes of administration, the authors consider fairly well established by the statistics. They add a theoretical argument. They contend that there is no certainty that serum introduced intrathecally can actually come in contact with the diseased nerve cells, or, if it does, that it can free them of the toxin. If the serum is introduced intravenously, it is at once diluted by the entire blood stream, and therefore its action at any one point in the nerve system must be very slight.

On the other hand, serum introduced subcutaneously or intramuscularly, is retained for a considerable length of time in a highly concentrated state, and therefore, if care is taken to inject it along the probable course of transit of the poison from the wound to the central nervous system, there is a fair chance of its acting directly upon the nerves by which the poison is absorbed. In this case, the long period of absorption, which has generally been considered an objection to these methods, may be the chief element in their success.

They therefore recommend the following procedure : " Should the wound be a single one, and especially if it is on a limb, we believe it would be sound practice to introduce antitoxin, both subcutaneously and intramuscularly, somewhere astride the path which the toxin must travel on its way to the spinal cord. To do this effectively it would appear, if our ideas are correct, better to divide the dose and inject portions on each surface of the limb, and also at different depths amid the muscles. Somewhere among these tissues we know the nerves run along which the poison is travelling, and the more evenly distributed our antitoxin the better would appear the chance that it may attain effective contact with the toxin and achieve our object ".

On the subject of dosage, they conclude that it is not so much the quantity used, as the certainty of contact in high concentration with the toxin. They think, however, that the daily injections given as advised above, should not fall below 10,000 units. If intrathecal injections are used to supplement them, these ought not to exceed the number of units that can be included in 10 or 15 cc.

In other respects the authors are mainly in accord with the recommendations of the Tetanus Committee. They find themselves unable to add any suggestions to help in diagnosis, but note that many of the signs mentioned by the Committee may lead to confusion with other wound infections.

